# Modeling the 3D genome of plants

**DOI:** 10.1080/19491034.2021.1927503

**Published:** 2021-05-31

**Authors:** Marco Di Stefano, Hans-Wilhelm Nützmann

**Affiliations:** a Institute of Human Genetics, Centre National de la Recherche Scientifique, University of Montpellier, Montpellier, France; b The Milner Centre for Evolution, Department of Biology and Biochemistry, University of Bath, Bath, UK

**Keywords:** Plants, 3D genome, chromosome modeling, polymer simulations, epigenome, Hi-C

## Abstract

Chromosomes are the carriers of inheritable traits and define cell function and development. This is not only based on the linear DNA sequence of chromosomes but also on the additional molecular information they are associated with, including the transcription machinery, histone modifications, and their three-dimensional folding. The synergistic application of experimental approaches and computer simulations has helped to unveil how these organizational layers of the genome interplay in various organisms. However, such multidisciplinary approaches are still rarely explored in the plant kingdom. Here, we provide an overview of our current knowledge on plant 3D genome organization and review recent efforts to integrate cutting-edge experiments from microscopy and next-generation sequencing approaches with theoretical models. Building on these recent approaches, we propose possible avenues to extend the application of theoretical modeling in the characterization of the 3D genome organization in plants.

## Introduction

The organization of chromosomes modulates the activity and efficiency of all DNA-related processes – from mitosis to DNA repair and from replication to gene expression [1–3]. Changes to the native structure of chromosomes by natural processes, such as mutation, transposition and recombination, or transgenic approaches can lead to drastic changes in the activity of these processes. In humans, this has been shown to result in severe disease phenotypes [4–6].

The effect of the genome on transcription can be divided into three interwoven elements. First, the genome is a one-dimensional object consisting of an array of nucleotides that define genes and regulatory sequences (e.g., promoters, enhancers, and insulators) on the DNA molecule [7]. Second, the DNA is locally wrapped around histones to form chromatin. Both DNA and histones can be decorated by a regulatory layer of proteins (e.g., transcription factors, mediators) or chemical groups, which affect how the DNA is read and used in the nucleus without changing the sequence. The mechanisms of action of these DNA regulators and their inheritability are active research topics in the fields of epigenomics and epigenetics. Third, the genome is organized in chromosomes which are the physical carriers of the genes, and have a complex and dynamic structural (3D) folding. When completely stretched out, chromosomes are centimeters in length [8]; however, within the eukaryotic nucleus, they occupy a highly limited space of few micrometers undergoing extreme compaction and compartmentalization. As a result, chromosome folding may bring functional sequence elements that are distant along the genomic sequence close to each other in the 3D space, thus, constituting a fundamental layer of gene regulation (Bonev and Cavalli 2016).

**Figure 1.**
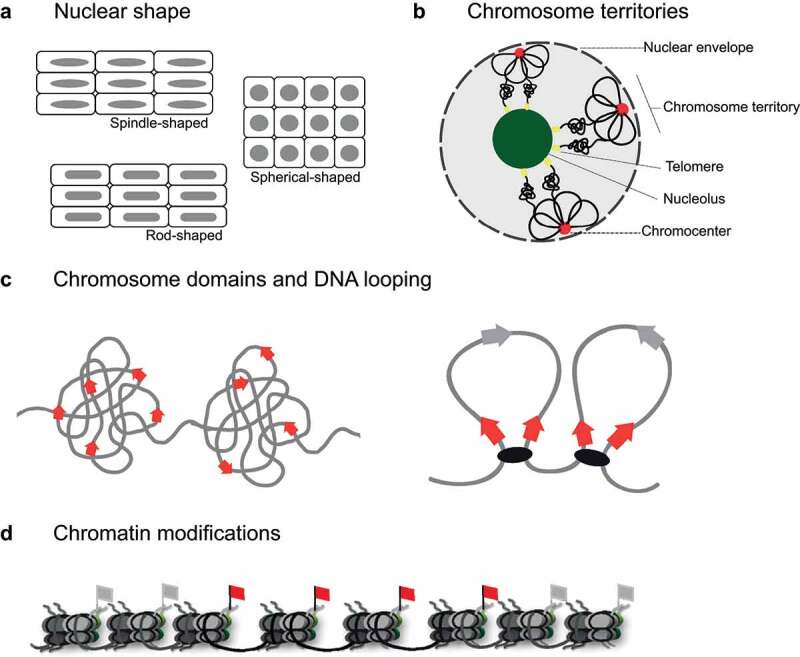
Schematic representation of key parameters in modeling of plant nuclear architecture. (a) Nuclear shape determines the available space for the genome. (b) Chromosome territories provide major attachments sites for chromosomes and determine broad chromosome architecture. Exemplar schematic of the *A. thaliana* rosette chromosome configuration presented. (c) Domains and DNA loops provide local chromosome environments for genomic areas with shared features. (d) Chromatin modiﬁcations (red and gray flags) determine characteristics of individual monomers in chromosomal polymer chains

Our current understanding of genome folding has been shaped by the introduction of high-resolution imaging and chromosome conformation capture (3 C) techniques, which have put on evidence a layered organization of chromosomes. This is constituted by a global chromosome arrangement into chromosome territories, and local folding into compartments (1–10 Mbp), topologically associating domains (TADs, 100 kb – 1 Mb) and loops (1 kb – 100 kb) [9–14]. Each layer exhibits a remarkable flexibility and inherent property to dynamically reorganize. Chromosomal regions may suffer changes in their compaction states from a highly condensed to a looser structure and may localize to different areas of the nucleus. Interestingly, this characterization of the genome’s 3D structure was possible thanks to synergistic experimental and theoretical approaches, which have allowed the analysis, interpretation, and modeling of the experimental data unveiling how the layers of the organization interplay with each other, and how the 3D genome relates to (epi)genetic features (Figure 1).

In plants, chromosome structure has historically been studied at microscopic level. Since 2012, chromosome conformation capture (3C) technology, which uses the likelihood of contacts between distant chromosomal sites to infer chromosome folding, and its high-throughput variant Hi-C have been implemented in the field of plant sciences and, in conjunction with high- and super-resolution microscopy, allowed major progress in the elucidation of the organization of chromosomes in the 3D space of plant nuclei [15]. Overall, plant chromosome organization follows the architectural principles observed in animals [15]. However, a set of unique features and significant heterogeneity across species has been noted in chromosomal organization of plants. An array of recent review articles provides a comprehensive overview of our current understanding of the organization of chromosomes in the 3D space of the plant nucleus [16–21]. Here, we will summarize the main characteristics of nuclear chromosome organization in plants, and introduce a conceptual framework for the application of theoretical chromosome modeling to expand our ability to interpret and understand the complexity in plant genome organization in the nuclear space.

## Elements of 3D chromosome architecture in plants

### Nuclear shape

Plant nuclei are characterized by a remarkable structural plasticity. In the model species *Arabidopsis thaliana* alone, nuclear form varies between spherical, spindle, oval, invaginated, flattened and rod-shaped [22–25]. The differences in shape are accompanied by variability in size, whereby cell size largely correlates with nuclear size [25]. In addition to cell-type-specific size differences, changes in nuclear size may occur in response to developmental and environmental conditions. For example, in plants, a decrease in nuclear size has been observed upon osmotic stress and seed dormancy, while the application of heat stress has been shown to be accompanied by an increase in nuclear size [26–30]. Components of the nucleoskeleton, cytoskeleton, nuclear envelope and nuclear pores as well as lamin-like structures adjacent to the nuclear envelope have been suggested to determine nuclear shape in plants [25,31–40]. The nuclear envelope and the associated lamin and lamin-like structures have been shown to play important roles in the topological organization of genomes and transcriptional activity in both animals and plants [41]. Large sections of chromosomes are associated with the nuclear periphery [42–44]. These so-called ’lamina-associated domains’ (LADs) and ‘plant lamina-associated domains’ (pLADs) are typically characterized by heterochromatic organization and low transcriptional activity [42,43,45]. Lamin and lamin-like proteins mediate the interaction between the nuclear periphery and chromatin. Loss of lamin-like CROWDED NUCLEIC (CRWN) proteins in *A. thaliana* has been shown to lead to drastic changes in chromatin organization [31,44,46].

### Territorial organization of chromosomes

In the interphase nucleus, individual eukaryotic chromosomes segregate into distinct regions [1,9]. This segregation into chromosome territories has been suggested to enable a functional compartmentalization of the nucleus [1]. In plants, chromosome territories have been identified in species of various genome sizes [22,47–49]. Within these territories, plant chromosomes often adopt distinct configurations. The relatively small genome of *A. thaliana* (~135 Mb) arranges into a so-called ‘rosette’ structure which is characterized by a core element consisting of heterochromatic chromocenters and emanating loops of euchromatic chromosome arms [47]. Larger plant genomes such as those of wheat, barley and oat are often organized in a ‘Rabl’ configuration [50,51]. Rabl chromosomal arrangements are characterized by the localization of centromeres and telomeres at opposite poles of the nucleus. Notably, these chromosomal configurations can be variable across plant tissues [51]. A third major structural configuration – the ‘bouquet’ – is associated with the early meiotic prophase and characterized by clustering of telomeres at the nuclear envelope [51–55].

### Chromosome compartments

Within chromosome territories, chromosomal regions can be broadly divided into active A and repressive B compartments [10]. Largely defined by chromatin marks and transcriptional activity, these compartments reflect the preferential interaction of active with active and inactive with inactive areas of the genome [10,56]. Plant genomes show a similar partitioning into active and inactive compartments [46,57,58]. Analyzed on whole chromosome-level, euchromatic chromosome arms represent active A compartments and centromeric as well as pericentromeric regions correspond to repressive B compartments [46,57]. Examined within chromosome arms only, a further partitioning into sub A and B compartments can be observed. Referred to as loose and closed structural domains (LSDs and CSDs) in *A. thaliana*, these sub A and B compartments separate euchromatic and heterochromatic regions within chromosome arms [46,57]. The distribution of sub-compartments along chromosomes shows tissue-specific dynamics and may change upon activation and repression of chromosomal regions [59–61].

### Local physical domains or TADs

TADs are organizational units of the 3D genome that show increased *within* contact frequency [11,62]. While TADs are hallmarks of the mammalian and the *Drosophila* 3D genome, plant genomes show a more diverse TAD and TAD-like organization. Species such as *A. thaliana* and *Arabidopsis lyrata* lack a traditional TAD pattern and TAD-like structures are limited to small and dispersed chromosomal regions [46,63,64]. In contrast, in other species, such as wheat, maize, rice and the liverwort *Marchantia polymorpha* a more prominent TAD patterning of their respective genomes can be observed [49,57,58,65]. However, these TADs do not always neighbor each other; instead, non-TAD areas may be located adjacent to TADs [65]. On plant chromosomes, heterochromatic DNA elements are often enriched within TADs while TAD boundaries are marked by active genes [49,57,65]. Concia and collaborators have introduced the term ICONS (intergenic condensed spacers) to describe the non-canonical genetic organization within these TADs [49]. TADs can be further classified into different categories depending on their decoration with chromatin marks and association with transcription factors [57,66]. Unlike in other plant species, genes within TADs of *M. polymorpha* show an increased tendency for co-expression [65].

### Chromatin loops

Chromatin loops describe short- and long-range interactions of chromosomal sites distant from each other on the linear sequence level. In plants, chromatin loops have been described in the context of distant regulatory site and promoter contacts, contacts between 5ʹ and 3ʹ ends of genes, and interactions of gene islands and heterochromatic islands scattered across the genome [46,67–75]. For example, high-resolution Hi-C analysis has identified a high prevalence of short-range loops in the *A. thaliana* genome [76]. Furthermore, by Chromatin Interaction Analysis by Paired-End Tag Sequencing (ChIA-Pet) and *in situ* digestion-ligation-only Hi-C (DLO-Hi-C) a widespread formation of gene-to-gene, promoter – promoter and promoter – distal regulatory site loops have been identified in maize and rice [69,70,74,77]. Here, loops predominantly span regions between 100 and 500 kb yet can connect DNA sites up to >2 Mb away from each other. Genes connected in gene-to-gene loops show a tendency for co-expression and are suggested to form spatial gene clusters in accordance with the concept of transcription factories as described below [49,71,78,79]. Gene – distal regulatory site loops in maize are established between single promoters or promoters of multiple genes and correlate with gene expression differences. Interestingly, such chromosomal loops partly overlap with intergenic quantitative trait loci in both maize and rice [69,70,74]. In addition to transcription factors and chromatin markings, long non-coding RNAs (lncRNA) have been implicated in chromatin loop formation. Two recent reports provide mechanistic insight into the interplay between lncRNA and chromatin loops in *A. thaliana*. Here, it is shown that the lncRNA *APOLO* is involved in the regulation of auxin-responsive genes and the lncRNA *MARS* in the abscisic acid-induced expression of a biosynthetic gene cluster. Thereby, *APOLO* interacts with LHP1, a homolog of the animal HP1 and component of the PcG complex, and associates with locally formed loops at multiple loci across the *A. thaliana* genome. The recognition of target sites by *APOLO* is suggested to be mediated by the formation of R-loops [80,81]. *MARS* interacts with LHP1 and modulates loop formation within the marneral biosynthetic gene cluster. Once expressed, *MARS* binds LHP1 and decoys LHP1 away from the cluster. The formation of this loop is abscisic acid driven and is suggested to connect an enhancer element with its target gene [82].

An intriguing characteristic of nuclear chromosome structure in *A. thaliana* is the so-called KNOT chromosome structure [46,63]. Here, chromosome regions of 50 to 150 kb in size and enriched in transposable elements form a strong network of intra- and interchromosomal contacts in the 3D nuclear space. Recent findings indicate that KNOT regions have the potential to reorganize and incorporate newly integrated DNA elements into the KNOT structure [83]. This process has been proposed as a mechanism of gene silencing of foreign DNA elements [83]. Interestingly, KNOT formation is altered in several chromatin mutant lines and shows organ-specific diversity [59,83,84]. A similar structure, called the ‘compact silent center’ (CSC), has been identified in rice genomes and shows similar potential for chromosomal reorganization in different cell-types [61,75]. In *M. polymorpha*, a network of chromosomal regions labeled with H3K27me3 and showing extensive long-range interaction has been suggested to resemble KNOT regions in *A. thaliana* and rice [65].

### Major structural units within the nucleus

Multiple major structural units can be identified in the nucleus in addition to the hierarchical units of chromosome territories, compartments and domains. These units anchor chromosomal regions and are of functional importance for chromosome processes such as transcription and replication. Structures like the nucleolus, chromocenters and telomeres are formed around major chromosomal sequence elements [21]. Other nuclear bodies, such as Cajal bodies and speckles, are associated with distinct epigenomic and transcriptional states of chromosomal regions [21].

#### Nucleolus

The nucleolus is the largest compartment in the nucleus. It is the site of ribosome biogenesis and is characterized by a high density of proteins [85]. In higher plants, the nucleolus is typically organized in a near-spherical shape that dynamically adapts form, size, and position within the nucleus according to the cell type, cell cycle phase, transcriptional activity and physiological state of the cell [86,87]. The nucleolus forms around active nucleolar organizer regions (NORs), tandem arrays of rRNA genes [88]. In addition to the NORs, a substantial fraction of the genome can dynamically associate with the nucleolus. These regions are collectively termed ‘nucleolus-associated chromatin domains’ (NADs) [89]. In humans, NADs comprise primarily gene-poor and heterochromatic chromosomal regions [89]. In *A. thaliana*, NADs contain actively transcribed rRNA genes, subtelomeric regions and hundreds of silenced genes [90]. It has been suggested that NAD composition is primarily defined by rRNA gene organization and transcriptional activity. Indeed, it has been observed that both loss of rRNA copies and changes in the rRNA expression state lead to changes in NAD composition [90–93]. The application of modest heat stress to *A. thaliana* seedlings results in reorganization of the nucleolus. This, however, is not associated with changes in NAD composition [94].

#### Chromocenters

Chromocenters are detectable as highly condensed chromosomal regions in the interphase nucleus. In *A. thaliana*, chromocenters are formed by heterochromatic centromeric and pericentromeric regions [47]. Here, chromocenters tend to be positioned at the nuclear periphery [22,47,57]. In Hi-C contact maps of *A. thaliana* chromosomes, chromocenters are characterized by strong interaction patterns [63]. Intra-centromeric and pericentromeric interactions are less pronounced in other plant species such as maize, tomato, rice and foxtail millet [57,58]. However, in genomes with Rabl configuration, significant enrichment for intercentromeric contacts are detectable [57]. In *A. thaliana,* intra-chromocenter interactions vary between tissues and plants grown under different environmental conditions. For example, heat stress is associated with reduced chromocenter interactions and a root-leaf comparison shows decreased chromocenter contacts in root nuclei [30,59,95]. Furthermore, chromocenter decondensation has been shown in *A. thaliana* mutants with reduced capacity for DNA methylation and histone H3 lysine 9 methylation [63,96]. Similar to chromocenters, so-called knobs are visible as condensed chromosomal areas in the interphase nucleus. However, in contrast to chromocenters, they are not associated with centromeres. Instead, they are composed of arrays of tandem repeats primarily positioned on chromosome arms [97–100]. Interestingly, circular chromosome conformation capture (4C) experiments that measure the genome-wide contact probabilities of a target site have shown an enrichment of interactions between the *hk4s* knob region and pericentromeres in *A. thaliana* [95]. The *hk4s* knob is derived from an inversion of a pericentromeric region and it is suggested that its 3D interactome reflects the original genome positioning of the knob [95].

#### Telomeres

Telomeres constitute the ends of chromosomes and are characterized by an array of repetitive elements. In both rosette and Rabl configurations, telomeres of different chromosomes are co-localized. In *A. thaliana* and sorghum, telomeres are positioned at the nucleolus and in species such as wheat and barley they are polarized to one side of the nucleus [47,50,52,90]. Nucleosome decoration and arrangement have been implicated in the conformational properties of chromocenter and telomeres in *A. thaliana*. For example, loss of the linker histone H1 has been associated with a global chromatin decondensation particularly pronounced for the pericentromeric chromosome regions [101]. Loss of H1 was further shown to be associated with more frequent interactions between telomeres and their re-positioning away from the nucleolus [102]. A similar pattern of enhanced chromocenter decondensation and telomere interactions has been observed in the chromatin remodeling mutant *morc6* [63,103].

#### Nuclear bodies

Nuclear bodies such as Cajal bodies, Polycomb bodies and transcription factories are detectable as small cytological structures interspersed throughout the nucleus [21,104]. The shape and formation of nuclear bodies is dependent on the developmental and physiological state of the individual cell. Typically, they provide microenvironments for specialized nuclear processes such as transcriptional regulation and DNA repair and are often enriched for distinct proteins [21,104].

Associated with the nucleolus, Cajal bodies contain components of the RNA processing machinery [105]. Variable in size and number across cell types, these subnuclear organelles play important roles in the processing of RNA species and ribonuclear proteins. In plants, Cajal bodies have been suggested to be involved in gene regulation, viral infections and the environmental stress response [106,107]. In animal systems, Cajal bodies have been implicated in genome organization and shown to associate with clusters of histone genes [105,108].

Polycomb bodies are enriched for Polycomb group (PcG) proteins. PcG proteins play major roles in the epigenetic silencing of genes and establish distinct foci in the interphase nucleus. Genomic regions bound by PcG proteins and marked with the histone modification H3K27me3 tend to cluster in the linear and three-dimensionally folded genome in both plants and animals [105,108–113]. Impaired PcG activity in *A. thaliana* results in reduced contact probability between H3K27me3 marked domains [59,63,114].

In contrast to polycomb bodies, transcription factories are associated with active transcription. Transcription factories are discrete nuclear foci that are composed of a transcriptional complex containing active RNA polymerase II. Linearly nearby genes as well as genes distant in *cis* and *trans* may be positioned within a single transcription factory [78,79]. Recent findings by Concia and collaborators (2020) suggest that transcription factories are also established in the 3D genome of wheat [49]. Such sub-nuclear co-localization of genes in transcription factories is proposed to facilitate co-ordinate expression of genes [49,115].

## Modeling 3D chromosome architecture

Together, classical experimentation and recent advances in microscopy and structural genomics have provided us with a solid knowledge base on the nuclear chromosome organization of plants. It is, however, worth noting that our current models of plant chromosome organization are so far, by large, lacking a generalized interpretation and a robust understanding of the key elements driving nuclear chromosome folding. In the following section, we introduce the latest development from structural (3D) computer modeling of chromosomes and highlight how numerical approaches have helped to analyze and interpret experimental results. Most applications of modeling have been aimed at animal species, but, notably, a recent application involved unveiling the constitutive mechanisms of the genome in *A. thaliana* [116]. Furthermore, we propose that modeling approaches could be extended to other plant species and help to unravel the specific complexity of their 3D genomes.

### Data-driven modeling of the 3D genome architecture

In the past, the development of new experimental techniques in structural genomics has been complemented by theoretical approaches aimed to generate three-dimensional (3D) models of the genomic region of interest. An important example was the introduction of the 3C technique, which determined the folding of yeast chromosome III [117]. More recently, the introduction of the single-cell Hi-C (scHi-C) [118] technique was complemented with the modeling of the cell-specific entire X chromosome at a resolution of 500 kb, which allowed correlation of the scHi-C data with results from FISH imaging. Also, the potential of new super-resolution imaging techniques has been accelerated by combined experimental and modeling approaches. Nir and collaborators showed that by integrating OligoSTORM (Stochastic Optical Reconstruction Microscopy) and OligoDNA-PAINT (Point Accumulation for Imaging in Nanoscale Topography) imaging with Hi-C interaction maps, it was possible to reconstruct the structure of active and repressive (A/B) compartments. This allowed a quantitative examination of the compartment-type dependent degree of entanglement, which was not immediately accessible from neither the images nor the interaction data [119].

The modeling approaches discussed so far are part of the so-called data-driven (top-down) modeling. The latter encompasses a plethora of strategies in which the 3D organization is directly inferred from experimental data [120]. These approaches typically follow four methodological steps:

#### Data collection

Source data for modeling approaches are produced contextually or gathered from repositories such as GEO (Gene Expression Omnibus) [121] and subsequently formatted and analyzed to make them usable for a modeling pipeline [122,123]. Examples of data which can be used are as follows: the shape and size of nuclei [124,125], the positions and the spatial distances between genomic loci in the nucleus [119], or the interactions counts measured in 3C-based experiments [124,126–128].

#### Data representation

The first important step of 3D genome modeling is to represent the chromatin fiber as a physical object (polymer) of consecutive particles (monomers). Most of the modeling strategies use spherical particles which, depending on the approach, can have all the same size and represent the bins of the experimental interaction map obtained in Hi-C experiments [122,127], or can have different sizes to describe TADs [128–130] and the regions probed during imaging experiments [131].

#### Model scoring

A mathematical function is defined to evaluate the consistency between each conformation of the models’ particles and the experimental data. The aim of this is to favor the 3D models that recapitulate the input data (spatial distances or contact propensities) and penalize the ones that are not compatible. The definition of this so-called scoring function is typically one of the most delicate tasks of the approach and requires significant trial-and-error, since an inaccurate score might lead to inadequate solutions. This is especially true for modeling based on Hi-C data, because the definition of the scoring function requires the transformation of the interaction counts into spatial distance restraints, which is typically a complex task.

#### Model sampling

The possible model conformations are sampled using Monte Carlo or molecular dynamics methods to explore as many solutions as possible compatible with the scoring function. Finally, the sampled structures are ranked based on the scoring function and the models optimally satisfying the imposed data-driven restraints are deemed the ones representing the input data and retained for further analysis [120,127].

Although data-driven approaches have been widely used in animals [120,132,133], applications to characterize the structural organization of plant genomes are limited. In rice, single-cell Hi-C (scHi-C) interaction maps have been used to obtain genome-wide models of eggs, sperm, unicellular, zygotes (Z) and mesophyll (M) cells [61]. The 3D models were instrumental in characterizing cell-specific features of chromosome compartments and telomere/centromere configurations. In particular, the 3D genomes of the eggs and unicellular zygotes were found to contain a ‘compact silent center’ (CSC) that is absent in sperm cells. CSC appears to be reorganized after fertilization, and may be involved in the regulation of zygotic genome activation [61].

**Figure 2.**
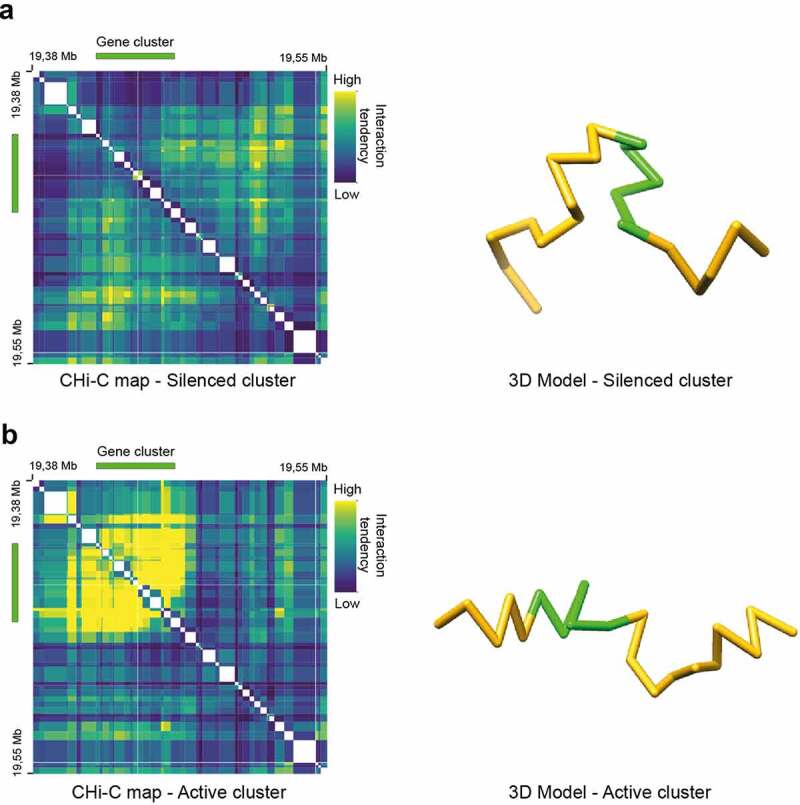
3D modeling of local chromosome conformation in *A. thaliana*. Left, 2D Capture Hi-C interaction maps of a 170 kb region in the *A. thaliana* genome that contains a gene cluster of co-expressed neighboring genes. Right, 3D modeling of the same 170 kb region using TADbit. In green, gene cluster. In (a), the gene cluster is silenced. In (b), the gene cluster is expressed. Adapted from Nützmann et al. [59]

In recent work [59], we used TADbit [122], one of the available modeling tools, to study the spatial organization of clusters of neighboring and co-expressed genes in *A. thaliana*. Using high-resolution capture Hi-C data as source data, our structural modeling of the major 3D domains associated with such a cluster indicated that the transcriptionally active cluster assumes a compact conformation in which the clustered genes are in spatial proximity. When transcriptionally silent, the gene cluster is more extended and incorporated into a chromatin loop, which brings the co-expressed genes in spatial proximity with a nearby region of unknown function (Figure 2) [59].

### Bottom-up modeling of the 3D genome: a lesson from animal species

Theoretical strategies in chromosome modeling also include bottom-up (hypothesis-driven) approaches. The latter aims to build predictive models that test mechanistic hypotheses derived from experimental observations. By comparing predictions of genome structure to experiments, the models allow to invalidate or consolidate the underlying assumed mechanisms and, more interestingly, to propose and guide new experiments to obtain further insight. Relying on computer simulations and theoretical arguments as their primary tools, bottom-up modeling takes advantage of experimental data to parametrize the models and to validate the obtained results. The ultimate goal of this approach is to provide simple testable rules that can contribute, even partially, to understand the complexity of genome architecture,

The application of bottom-up modeling helped to propose and test several hypotheses on the passive and active physical mechanisms regulating the structural organization of genomes at different scales (for references see for example [134–136]).

#### Territorial organization of chromosomes

At the scale of entire chromosomes, polymer physics arguments and computer simulations hypothesized that chromosomes might organize as unknotted and unentangled crumpled or fractal globules [10,134,137,138], which can recapitulate the average spatial organization of chromosomes from imaging [139] and Hi-C measurements [10]. Furthermore, building on the analogy between ring and long confined polymers, physics arguments can recapitulate the formation of chromosome territories in interphase [134,138,140,141].

#### Chromosome compartments

Within chromosome territories, bottom-up approaches have suggested that chromosome compartmentalization might be stabilized by epigenomic-driven interactions [135,142–144]. Chromatin domains with the same epigenomic marks are proposed to interact with each other. The central idea is that phase-separation mediated by proteins, shown *in vitro* for heterochromatin protein 1 (HP1) [145,146], might also occur *in vivo*, leading to chromatin compartmentalization. The first study in this field focussed on *Drosophila melanogaster* [135]. It showed that block copolymer models built from the epigenomic landscape reproduce the formation of chromatin domains found in Hi-C interaction maps [62]. Additionally, these models suggested that epigenomic-driven chromosome domains are multi-stable as they can form and disassemble over time as well as interact dynamically with each other.

#### Local physical domains or TADs

The formation of physical domains or topologically associating domains [11,62,147] can be recapitulated by different mechanisms, including DNA supercoiling [148], loop-extrusion via active or passive mechanisms [136,149,150], or transcription factor-mediated contacts [151]. At the local scale, where promoter-enhancer contacts occur, the string-and-binders polymer model [152] has been employed to dissect the folding at several loci, such as *Xist* and *HoxB* [153,154]. In particular, the loop extrusion mechanism proposed a role for insulating proteins (CCCTC-binding factor or CTCF) and for proteins actively extruding chromatin (Cohesin). Interestingly, CTCF encoding genes are absent in plants and no functionally related proteins have been identified to date [19,155]. Cohesin proteins have been shown to be essential for chromosome pairing and meiosis in plants [156,157]. The role for cohesins in territory or domain formation in plant interphase nuclei, however, remains unknown.

### Bottom-up modeling in plants

In plant species, a limited set of modeling approaches have been proposed so far. For example, Pecinka and collaborators developed models of *A. thaliana* chromosomes at the resolution of 1Mb per particle and used simulations to test whether the association of chromosome territories (CTs) in interphase nuclei could be ascribed to random chromosome pairing [22]. Specifically, chromosomes were organized initially as linear rods and allowed to decondense inside confined environments of different shapes and sizes to mimic the diverse nuclei until the available space is filled uniformly [22]. Interestingly, the models demonstrated that chromosome pairing could be ascribed to random association even though some chromosomes had high association rates in images (e.g., chromosomes 1, 3 and 5 were seen to associate in up to 70% of the spherical nuclei).

More recently, the genome of *A. thaliana* was also explored by polymer-based modeling [158]. In particular, the authors tested which chromosome topology could recapitulate the positioning of chromocenters and the nucleolus at the periphery and center of the nucleus, respectively. Interestingly, the models suggested that only a chromosomal rosette conformation could recover the expected nuclear positionings. However, none of the tested models was able to reproduce the association between chromocenters. This suggested that additional mechanisms that were not implemented in the models play critical roles in chromocenter associations [158].

**Figure 3.**
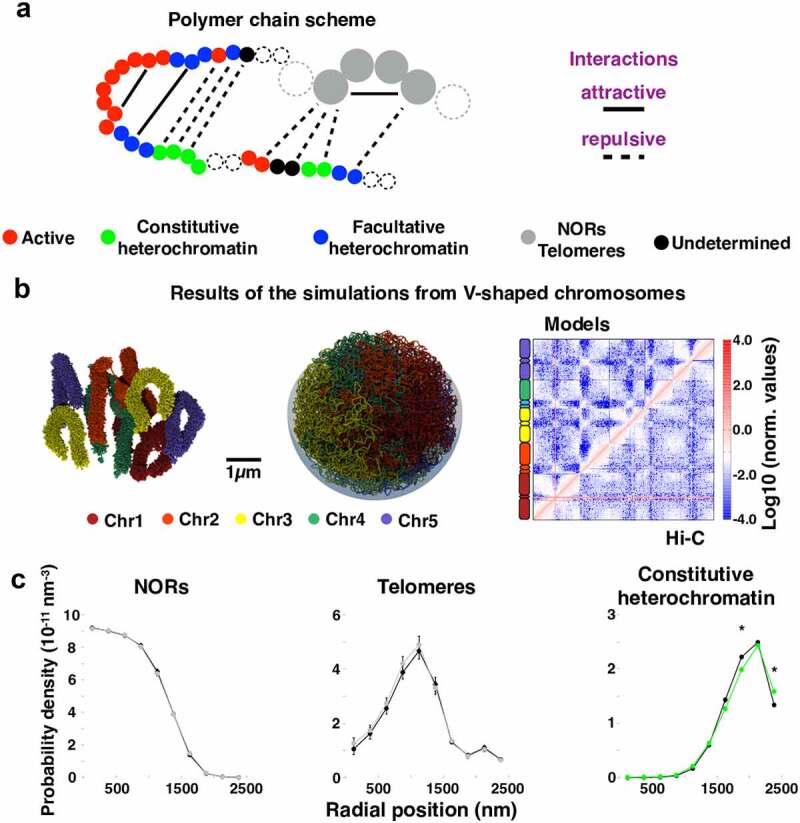
Epigenomic-driven models of the *A. thaliana* genome. (a) Polymer models of the *A. thaliana* chromosomes are decorated with epigenomic-driven interactions among active regions, constitutive and facultative heterochromatin, nucleolar organizing regions (NORs), telomeres, and undetermined chromatin. (b) Chromosomes are initially organized as V-shaped objects and, after molecular dynamics simulations in which the epigenomic-driven interactions are enforced, the system reaches a steady-state conformation where chromosomes spread within the spherical model nucleus. The contact maps computed on the models recapitulate experimental (Hi-C) data [76]. (c) The radial positioning of epigenomic regions in the optimal-interaction system is compared with a reference case (black curves) in which all interactions are dropped but the ones involving NORs and telomeres. The model predictions recapitulate what is expected from imaging experiments: the nucleolus (NORs) mainly occupies the nuclear center, telomeres localize at the nuclear periphery (~1400 nm from the nuclear center), and constitutive heterochromatin is significantly enriched at the most peripheral shell of the model nucleus (two-sided Wilcoxon test *p*-value <0.0001). Adapted from Di Stefano et al. [116]

To deepen the understanding of the 3D genome organization in *A. thaliana*, we recently applied bottom-up modeling approaches integrating data on the length of the chromosomes and the nucleolar-organizing regions, the size and shape of the nucleus and the nucleolus, as well as epigenomic features [116]. Specifically, we incorporated the observation that chromosome regions hosting the same histone marks tend to co-localize in the 3D space forming compartments. Hence, we partitioned the genome in epigenomic states by looking at the enrichment in histone marks: active (A, enriched in H3K4me1/3 and H3K27ac), constitutive heterochromatin (CH, enriched in H3K9me3), facultative heterochromatin or polycomb-like (FH, enriched in H3K27me3), and undetermined (non-enriched) chromatin. We next tested several possible physical interactions between beads of the same or different epigenomic state and found that to optimize similarity with Hi-C contact patterns some interactions were needed (Figure 3(a)). These included attractive interactions between the nucleolar organizing regions on chromosomes 2 and 4, repulsive interactions between constitutive heterochromatin and the other chromatin states, as well as self-attractive interactions of active and polycomb-like domains. Additionally, to maximize the correlation with the Hi-C data, we had to organize the initial chromosome conformations as V-shaped objects (Figure 3(b)). The latter is a sign of an interesting ‘structural memory’: chromosomes in interphase are partially reminiscent of the overall reorganization they undergo during anaphase, when the two copies of each chromosome during cell division are pulled centromere first to opposite poles of the mother cells. These mechanisms allowed the recovery of several experimental features including the genome-wide Hi-C interaction pattern, the formation of the nucleolus in the nuclear center, the positioning of telomeres at the nucleolar periphery, and the enrichment of constitutive heterochromatin at the nuclear periphery (Figure 3(b,c)).

## Perspectives

So far, approaches of theoretical modeling have not widely been employed in studies investigating plant genome architecture. To expand their application in top-down or bottom-up modeling strategies of plant genomes, a set of minimal data must be available for the species or condition of interest. Specifically, an estimation of chromosome length and ploidy state for the investigated cells is essential for model parameterization and for accuracy and reliability of the quantitative predictions. Additionally, information regarding size and shape of the nucleus will benefit the confinement of genome-wide models. Epigenomic and transcriptomic data will enable the development of polymer models with beads of individual interaction characteristics. Importantly, it should be noted that a subset of data could be left aside for initial model production and instead be used for model validation.

Several intriguing open questions in plant genome organization could be addressed using modeling approaches. It is feasible to expand the analyses performed in *A. thaliana* [116,158] to predict chromosome architecture across different cell-types and plant species. An obvious direction would be to develop simulations that merge larger experimental datasets, covering epigenomics, Hi-C, and microscopy, with different nuclear shapes and sizes to study their impact on genome structural organization. Another route may be to simulate different chromosome length as well as positioning and size of centromeres to study their impact on Rabl, rosette or bouquet-like chromosomes organization. This could be combined with experimental data from plant species with diverse chromosome sizes and centromere positioning. An important aspect that emerged from our previous work is the importance of ‘structural memory’ in *A. thaliana* chromosome organization and how the preferred V-shape of chromosomes is ultimately related to the presence of a unique centromere, which corresponds to the kinetochore. It would be of great interest to model chromosomes with multiple centromeres [159] and characterize their role in genome-wide organization. Furthermore, our current modeling approaches have been unable to integrate KNOT structures in the optimal models for *A. thaliana* genome organization. Refinement in the preconditioning of these models and incorporation of novel experimental data on genomic features of KNOTs and their short or long-range interactions may enable us to elucidate their functional and structural constraints within the nucleus.

In plants, complex ploidy states and the extreme variance in genome sizes provide further tantalizing routes for chromosome modeling. Moreover, simulating the integration of novel DNA elements, such as transposons, introgressions and transgenes, into plant genomes will benefit our abilities to predict their impact on native genome organization. Altogether, these approaches may expand our fundamental understanding of eukaryotic genome organization and improve gene technology and breeding processes.

A wider adoption of modeling approaches in the plant kingdom will be favored by an expansion of existing or the establishment of novel databases that host genomics, epigenomics and microscopic data for various plant species with unified quality standards and nomenclature. This would enable a rapid input of data into generated simulation pipelines and significantly facilitate the development of optimized models.

## Conclusions

Here, we reviewed essential aspects of plant chromosome organization and recent efforts of the experimental and modeling community to unveil the principles regulating 3D genome organization, and its interplay with the epigenome. Overall, we believe that further synergistic studies integrating experiments and modeling approaches will advance our understanding of the rules and constraints of plant chromosome organization. In our view, these studies should aim both to apply the tools developed and used to study the 3D genome in animals, but also to establish new modeling strategies to help address open questions in plant chromosome organization. Ultimately, this will allow us to build unified models of genome organization in the eukaryotic nucleus.

## References

[1] Mehta IS, Kulashreshtha M, Chakraborty S, et al. Chromosome territories reposition during DNA damage-repair response. Genome Biol. 2013;14(R135):R135. 24330859 10.1186/gb-2013-14-12-r135 PMC4062845

[2] Bonev B, Cavalli G. Organization and function of the 3D genome. Nat. Rev. Genet. 2016;17(11):661–678. 27739532 10.1038/nrg.2016.112

[3] Sima J, Chakraborty A, Dileep V, *et al*. Identifying *cis* elements for spatiotemporal control of mammalian DNA replication. Cell. 2019;176(4):816–830.e18. 30595451 10.1016/j.cell.2018.11.036 PMC6546437

[4] Hnisz D, Weintraub AS, Day DS, *et al*. Activation of proto-oncogenes by disruption of chromosome neighborhoods. Science. 2016;351(6280):1454–1458. 26940867 10.1126/science.aad9024 PMC4884612

[5] Valton A-L, Dekker J. TAD disruption as oncogenic driver. Curr Opin Genet Dev. 2016;36:34–40. 27111891 10.1016/j.gde.2016.03.008 PMC4880504

[6] Lupiáñez DG, Spielmann M, Mundlos S. Breaking TADs: how alterations of chromatin domains result in disease. Trends Genet. 2016;32(4):225–237. 26862051 10.1016/j.tig.2016.01.003

[7] Andersson R, Sandelin A. Determinants of enhancer and promoter activities of regulatory elements. Nat. Rev. Genet. 2020;21(2):71–87. 31605096 10.1038/s41576-019-0173-8

[8] Rosa A, Zimmer C. Chapter nine - computational models of large-scale genome architecture. In: Hancock R, Jeon KW, editors. International review of cell and molecular biology. San Diego: Academic Press; 2014. p. 275–349. 10.1016/B978-0-12-800046-5.00009-6 24380598

[9] Cremer T, Cremer C. Chromosome territories, nuclear architecture and gene regulation in mammalian cells. Nat. Rev. Genet. 2001;2(4):292–301. 11283701 10.1038/35066075

[10] Lieberman-Aiden E, Van Berkum NL, Williams L, *et al*. Comprehensive mapping of long-range interactions reveals folding principles of the human genome. Science. 2009;326(5950):289–293. 19815776 10.1126/science.1181369 PMC2858594

[11] Dixon JR, Selvaraj S, Yue F, *et al*. Topological domains in mammalian genomes identified by analysis of chromatin interactions. Nature. 2012;485(7398):376–380. 22495300 10.1038/nature11082 PMC3356448

[12] Rao SSP, Huntley M, Durand N, *et al*. A 3D map of the human genome at kilobase resolution reveals principles of chromatin looping. Cell. 2014;159(7):1665–1680. 25497547 10.1016/j.cell.2014.11.021 PMC5635824

[13] Ou HD, Phan S, Deerinck TJ, *et al*. ChromEMT: visualizing 3D chromatin structure and compaction in interphase and mitotic cells. Science. 2017;357(6349):eaag0025. 28751582 10.1126/science.aag0025 PMC5646685

[14] Szabo Q, Donjon A, Jerković I, *et al*. Regulation of single-cell genome organization into TADs and chromatin nanodomains. Nat. Genet. 2020;52(11):1151–1157. 33077913 10.1038/s41588-020-00716-8 PMC7610512

[15] Rowley MJ, Corces VG. Organizational principles of 3D genome architecture. Nat. Rev. Genet. 2018;19(12):789–800. 30367165 10.1038/s41576-018-0060-8 PMC6312108

[16] Pontvianne F, Grob S. Three-dimensional nuclear organization in *Arabidopsis thaliana*. J. Plant Res. 2020;133(4):479–488. 32240449 10.1007/s10265-020-01185-0

[17] Huang Y, Rodriguez-Granados NY, Latrasse D, *et al*. The matrix revolutions: towards the decoding of the plant chromatin three-dimensional reality. J. Exp. Bot. 2020;71(17):5129–5147. 32639553 10.1093/jxb/eraa322

[18] Pontvianne F, Liu C. Chromatin domains in space and their functional implications. Curr Opin Plant Biol. 2020;54:1–10. 31881292 10.1016/j.pbi.2019.11.005

[19] Dong P, Tu X, Liang Z, et al. Plant and animal chromatin three-dimensional organization: similar structures but different functions. J Exp Bot. 2020;71(17):5119–5128. 32374833 10.1093/jxb/eraa220

[20] Ouyang W, Xiong D, Li G, et al. Unraveling the 3D genome architecture in plants: present and future. Mol Plant. 2020;13(12):1676–1693. 33065269 10.1016/j.molp.2020.10.002

[21] Santos AP, Gaudin V, Mozgová I, *et al*. Tidying-up the plant nuclear space: domains, functions, and dynamics. J Exp Bot. 2020;71:5160–5178. 32556244 10.1093/jxb/eraa282 PMC8604271

[22] Pecinka A, Schubert V, Meister A, *et al*. Chromosome territory arrangement and homologous pairing in nuclei of *Arabidopsis thaliana* are predominantly random except for NOR-bearing chromosomes. Chromosoma. 2004;113(5):258–269. 15480725 10.1007/s00412-004-0316-2

[23] Chytilova E. Green fluorescent protein targeted to the nucleus, a transgenic phenotype useful for studies in plant biology. Ann Bot. 1999;83(6):645–654.

[24] Chytilova E, Macas J, Sliwinska E, *et al*. Nuclear dynamics in *Arabidopsis thaliana*. Mol Biol Cell. 2000;11(8):2733–2741. 10930466 10.1091/mbc.11.8.2733 PMC14952

[25] Meier I, Griffis AH, Groves NR, et al. Regulation of nuclear shape and size in plants. Curr Opin Cell Biol. 2016;40:114–123. 27030912 10.1016/j.ceb.2016.03.005

[26] Kater JM. A cytological study of dormancy in the seed of *Phaseolus vulgaris*. Ann Bot. 1927;os-41(4):629–642.

[27] Middendorf FG. Cytology of dormancy in *Phaseolus* and *Zea*. Bot Gaz. 1939;100(3):485–499.

[28] Finan JD, Guilak F. The effects of osmotic stress on the structure and function of the cell nucleus. J. Cell. Biochem. 2010;109(3):460–467. 20024954 10.1002/jcb.22437 PMC3616882

[29] Van Zanten M, Koini MA, Geyer R, *et al*. Seed maturation in *Arabidopsis thaliana* is characterized by nuclear size reduction and increased chromatin condensation. Proc. Natl. Acad. Sci. U. S. A. 2011;108(50):20219–20224. 22123962 10.1073/pnas.1117726108 PMC3250172

[30] Sun L, Jing Y, Liu X, *et al*. Heat stress-induced transposon activation correlates with 3D chromatin organization rearrangement in *Arabidopsis*. Nat Commun. 2020;11(1886):1–13. 10.1038/s41467-020-15809-5 PMC7170881 32312999

[31] Wang H, Dittmer TA, Richards EJ. *Arabidopsis* CROWDED NUCLEI (CRWN) proteins are required for nuclear size control and heterochromatin organization. BMC Plant Biol. 2013;13(1):200. 24308514 10.1186/1471-2229-13-200 PMC3922879

[32] Dittmer TA, Stacey NJ, Sugimoto-Shirasu K, et al. Little nuclei genes affecting nuclear morphology in *Arabidopsis thaliana*. Plant Cell. 2007;19(9):2793–2803. 17873096 10.1105/tpc.107.053231 PMC2048703

[33] Sakamoto Y, Takagi S. LITTLE NUCLEI 1 and 4 regulate nuclear morphology in *Arabidopsis thaliana*. Plant Cell Physiol. 2013;54(4):622–633. 23396599 10.1093/pcp/pct031

[34] Zhou X, Graumann K, Evans DE, et al. Novel plant SUN-KASH bridges are involved in RanGAP anchoring and nuclear shape determination. J. Cell Biol. 2012;196(2):203–211. 22270916 10.1083/jcb.201108098 PMC3265956

[35] Zhou X, Groves NR, Meier I. Plant nuclear shape is independently determined by the SUN-WIP-WIT2-myosin XI-i complex and CRWN1. Nucleus. 2015;6(2):144–153. 25759303 10.1080/19491034.2014.1003512 PMC4615252

[36] Graumann K, Vanrobays E, Tutois S, *et al*. Characterization of two distinct subfamilies of SUN-domain proteins in *Arabidopsis* and their interactions with the novel KASH-domain protein AtTIK. J. Exp. Bot. 2014;65(22):6499–6512. 25217773 10.1093/jxb/eru368

[37] Tamura K, Iwabuchi K, Fukao Y, *et al*. Myosin XI-i links the nuclear membrane to the cytoskeleton to control nuclear movement and shape in *Arabidopsis*. Curr. Biol. 2013;23(18):1776–1781. 23973298 10.1016/j.cub.2013.07.035

[38] Heslop-Harrison J, Heslop-Harrison Y. Organelle movement and fibrillar elements of the cytoskeleton in the angiosperm pollen tube. Sexual Plant Reprod. 1988;1(1). DOI: 10.1007/BF00227017.

[39] Tamura K, Hara-Nishimura I. Involvement of the nuclear pore complex in morphology of the plant nucleus. Nucleus. 2011;2(3):168–172. 21818409 10.4161/nucl.2.3.16175 PMC3149876

[40] Goto C, Tamura K, Fukao Y, et al. The novel nuclear envelope protein KAKU4 modulates nuclear morphology in *Arabidopsis*. Plant Cell. 2014;26(5):2143–2155. 24824484 10.1105/tpc.113.122168 PMC4079374

[41] Van Steensel B, Belmont AS. Lamina-associated domains: links with chromosome architecture, heterochromatin, and gene repression. Cell. 2017;169(5):780–791. 28525751 10.1016/j.cell.2017.04.022 PMC5532494

[42] Guelen L, Pagie L, Brasset E, *et al*. Domain organization of human chromosomes revealed by mapping of nuclear lamina interactions. Nature. 2008;453(7197):948–951. 18463634 10.1038/nature06947

[43] Bi X, Cheng Y-J, Hu B, *et al*. Nonrandom domain organization of the *Arabidopsis* genome at the nuclear periphery. Genome Res. 2017;27(7):1162–1173. 28385710 10.1101/gr.215186.116 PMC5495068

[44] Hu B, Wang N, Bi X, *et al*. Plant lamin-like proteins mediate chromatin tethering at the nuclear periphery. Genome Biol. 2019;20(87). DOI:10.1186/s13059-019-1694-3. PMC6492433 31039799

[45] Akhtar W, De jong J, Pindyurin A, *et al*. Chromatin position effects assayed by thousands of reporters integrated in parallel. Cell. 2013;154(4):914–927. 23953119 10.1016/j.cell.2013.07.018

[46] Grob S, Schmid MW, Grossniklaus U. Hi-C analysis in *Arabidopsis* identifies the KNOT, a structure with similarities to the flamenco locus of *Drosophila*. Mol Cell. 2014;55(5):678–693. 25132176 10.1016/j.molcel.2014.07.009

[47] Fransz P, De Jong JH, Lysak M, et al. Interphase chromosomes in *Arabidopsis* are organized as well defined chromocenters from which euchromatin loops emanate. Proc. Natl. Acad. Sci. U. S. A. 2002;99(22):14584–14589. 12384572 10.1073/pnas.212325299 PMC137926

[48] Berr A, Pecinka A, Meister A, *et al*. Chromosome arrangement and nuclear architecture but not centromeric sequences are conserved between *Arabidopsis thaliana* and Arabidopsis lyrata. Plant J. 2006;48(5):771–783. 17118036 10.1111/j.1365-313X.2006.02912.x

[49] Concia L, Veluchamy A, Ramirez-Prado JS, *et al*. Wheat chromatin architecture is organized in genome territories and transcription factories. Genome Biol. 2020;21(104). DOI:10.1186/s13059-020-01998-1. PMC7189446 32349780

[50] Němečková A, Koláčková V, Vrána J, et al. DNA replication and chromosome positioning throughout the interphase in three-dimensional space of plant nuclei. J. Exp. Bot. 2020;71(20):6262–6272. 32805034 10.1093/jxb/eraa370

[51] Dong F, Jiang J. Non-Rabl patterns of centromere and telomere distribution in the interphase nuclei of plant cells. Chromosome Res. 1998;6(7):551–558. 9886774 10.1023/a:1009280425125

[52] Tiang C-L, He Y, Pawlowski WP. Chromosome organization and dynamics during interphase, mitosis, and meiosis in plants. Plant Physiol. 2012;158(1):26–34. 22095045 10.1104/pp.111.187161 PMC3252114

[53] Scherthan H. A bouquet makes ends meet. Nat. Rev. Mol. Cell Biol. 2001;2(8):621–627. 11483995 10.1038/35085086

[54] Harper L, Golubovskaya I, Cande WZ. A bouquet of chromosomes. J Cell Sci. 2004;117(18):4025–4032. 15316078 10.1242/jcs.01363

[55] Cowan CR, Carlton PM, Cande WZ. Reorganization and polarization of the meiotic bouquet-stage cell can be uncoupled from telomere clustering. J. Cell Sci. 2002;115(19):3757–3766. 12235286 10.1242/jcs.00054

[56] Stevens TJ, Lando D, Basu S, *et al*. 3D structures of individual mammalian genomes studied by single-cell Hi-C. Nature. 2017;544(7648):59–64. 28289288 10.1038/nature21429 PMC5385134

[57] Dong P, Tu X, Chu P-Y, *et al*. 3D chromatin architecture of large plant genomes determined by local A/B compartments. Mol Plant. 2017;10(12):1497–1509. 29175436 10.1016/j.molp.2017.11.005

[58] Liu C, Cheng Y-J, Wang J-W, et al. Prominent topologically associated domains differentiate global chromatin packing in rice from *Arabidopsis*. Nat Plants. 2017;3(9):742–748. 28848243 10.1038/s41477-017-0005-9

[59] Nützmann H-W, Doerr D, Ramírez-Colmenero A, *et al*. Active and repressed biosynthetic gene clusters have spatially distinct chromosome states. Proc. Natl. Acad. Sci. U. S. A. 2020;117(24):13800–13809. 32493747 10.1073/pnas.1920474117 PMC7306824

[60] Dong P, Tu X, Li H, *et al*. Tissue-specific Hi-C analyses of rice, foxtail millet and maize suggest non-canonical function of plant chromatin domains. J. Integr. Plant Biol. 2020;62(2):201–217. 30920762 10.1111/jipb.12809

[61] Zhou S, Jiang W, Zhao Y, et al. Single-cell three-dimensional genome structures of rice gametes and unicellular zygotes. Nat Plants. 2019;5(8):795–800. 31332313 10.1038/s41477-019-0471-3

[62] Sexton T, Yaffe E, Kenigsberg E, *et al*. Three-dimensional folding and functional organization principles of the *Drosophila* genome. Cell. 2012;148(3):458–472. 22265598 10.1016/j.cell.2012.01.010

[63] Feng S, Cokus S, Schubert V, *et al*. Genome-wide Hi-C analyses in wild-type and mutants reveal high-resolution chromatin interactions in Arabidopsis. Mol Cell. 2014;55(5):694–707. 25132175 10.1016/j.molcel.2014.07.008 PMC4347903

[64] Zhu W, Hu B, Becker C, *et al*. Altered chromatin compaction and histone methylation drive non-additive gene expression in an interspecific *Arabidopsis* hybrid. Genome Biol. 2017;18(1). DOI:10.1186/s13059-017-1281-4. PMC5568265 28830561

[65] Montgomery SA, Tanizawa Y, Galik B, *et al*. Chromatin organization in early land plants reveals an ancestral association between H3K27me3, transposons, and constitutive heterochromatin. Curr. Biol. 2020;30(4):573–588.e7. 32004456 10.1016/j.cub.2019.12.015 PMC7209395

[66] Karaaslan ES, Wang N, Faiß N, *et al*. Marchantia TCP transcription factor activity correlates with three-dimensional chromatin structure. Nat Plants. 2020;6(10):1250–1261. 32895530 10.1038/s41477-020-00766-0

[67] Crevillén P, Sonmez C, Wu Z, et al. A gene loop containing the floral repressor *FLC* is disrupted in the early phase of vernalization. EMBO J. 2013;32(1):140–148. 23222483 10.1038/emboj.2012.324 PMC3545306

[68] Guo L, Cao X, Liu Y, *et al*. A chromatin loop represses *W**USCHEL* expression in *Arabidopsis*. Plant J. 2018;94(6):1083–1097. 29660180 10.1111/tpj.13921

[69] Li E, Liu H, Huang L, *et al*. Long-range interactions between proximal and distal regulatory regions in maize. Nat Commun. 2019;10(2633):1–14. 10.1038/s41467-019-10603-4 PMC6572780 31201330

[70] Peng Y, Xiong D, Zhao L, *et al*. Chromatin interaction maps reveal genetic regulation for quantitative traits in maize. Nat. Commun. 2019;10(2632). DOI:10.1038/s41467-019-10602-5. PMC6572838 31201335

[71] Wang H-LV, Chekanova JA. Novel mRNAs 3′ end-associated *cis*-regulatory elements with epigenomic signatures of mammalian enhancers in the *Arabidopsis* genome. RNA. 2019;25(10):1242–1258. 31311821 10.1261/rna.071209.119 PMC6800480

[72] H. Wang, et al. Connects enhancer-promoter looping and MYC2-dependent activation of jasmonate signalling Nat Plants . 2019;5:616–625. 31182849 10.1038/s41477-019-0441-9

[73] Louwers M, Bader R, Haring M, *et al*. Tissue- and expression level–specific chromatin looping at maize *b1* epialleles. Plant Cell. 2009;21(3):832–842. 19336692 10.1105/tpc.108.064329 PMC2671708

[74] Zhao L, Wang S, Cao Z, *et al*. Chromatin loops associated with active genes and heterochromatin shape rice genome architecture for transcriptional regulation. Nat Commun. 2019;10(3640):1–13. 10.1038/s41467-019-11535-9 PMC6692402 31409785

[75] Dong Q, Li N, Li X, *et al*. Genome-wide Hi-C analysis reveals extensive hierarchical chromatin interactions in rice. Plant J. 2018;94(6):1141–1156. 29660196 10.1111/tpj.13925

[76] Liu C, Wang C, Wang G, *et al*. Genome-wide analysis of chromatin packing in *Arabidopsis thaliana* at single-gene resolution. Genome Res. 2016;26(8):1057–1068. . 27225844 10.1101/gr.204032.116 PMC4971768

[77] Sun Y, Dong L, Zhang Y, *et al*. 3D genome architecture coordinates trans and *cis* regulation of differentially expressed ear and tassel genes in maize. Genome Biol. 2020;21(143). DOI:10.1186/s13059-020-02063-7. PMC7296987 32546248

[78] Schoenfelder S, Sexton T, Chakalova L, *et al*. Preferential associations between co-regulated genes reveal a transcriptional interactome in erythroid cells. Nat. Genet. 2010;42(1):53–61. 20010836 10.1038/ng.496 PMC3237402

[79] Osborne CS, Chakalova L, Brown KE, *et al*. Active genes dynamically colocalize to shared sites of ongoing transcription. Nat. Genet. 2004;36(10):1065–1071. 15361872 10.1038/ng1423

[80] Ariel F, Jegu T, Latrasse D, *et al*. Noncoding transcription by alternative RNA polymerases dynamically regulates an auxin-driven chromatin loop. Mol Cell. 2014;55(3):383–396. 25018019 10.1016/j.molcel.2014.06.011

[81] Ariel F, Lucero L, Christ A, *et al*. R-Loop mediated *trans* action of the *APOLO* long noncoding RNA. Mol Cell. 2020;77(5):1055–1065.e4. 31952990 10.1016/j.molcel.2019.12.015

[82] Roulé T, Ariel F, Hartmann C, *et al*. The lncRNA *MARS* modulates the epigenetic reprogramming of the marneral cluster in response to ABA. DOI:10.1101/2020.08.10.236562 35150931

[83] Grob S, Grossniklaus U. Invasive DNA elements modify the nuclear architecture of their insertion site by KNOT-linked silencing in *Arabidopsis thaliana*. Genome Biol. 2019;20(120). DOI:10.1186/s13059-019-1722-3 PMC6560877 31186073

[84] Yadav VK, Santos-González J, Köhler C. INT-Hi-C reveals distinct chromatin architecture in endosperm and leaf tissues of *Arabidopsis*. Nucleic Acids Res. 2021;49(8):4371–4385. 33744975 10.1093/nar/gkab191 PMC8096224

[85] Shaw PJ, Jordan EG. The nucleolus. Annu Rev Cell Dev Biol. 1995;11(1):93–121. 8689574 10.1146/annurev.cb.11.110195.000521

[86] Stępiński D. Functional ultrastructure of the plant nucleolus. Protoplasma. 2014;251(6):1285–1306. 24756369 10.1007/s00709-014-0648-6 PMC4209244

[87] Shaw P, Brown J. Nucleoli: composition, function, and dynamics. Plant Physiol. 2012;158(1):44–51. 22082506 10.1104/pp.111.188052 PMC3252080

[88] McStay B. Nucleolar organizer regions: genomic “dark matter” requiring illumination. Genes Dev. 2016;30(14):1598–1610. 27474438 10.1101/gad.283838.116 PMC4973289

[89] Van Koningsbruggen S, Gierliński M, Schofield P, *et al*. High-resolution whole-genome sequencing reveals that specific chromatin domains from most human chromosomes associate with nucleoli. Mol Biol Cell. 2010;21(21):3735–3748. 20826608 10.1091/mbc.E10-06-0508 PMC2965689

[90] Pontvianne F, Carpentier M-C, Durut N, *et al*. Identification of nucleolus-associated chromatin domains reveals a role for the nucleolus in 3D organization of the *A.* *thaliana* genome. Cell Rep. 2016;16(6):1574–1587. 27477271 10.1016/j.celrep.2016.07.016 PMC5279810

[91] Picart-Picolo A, Grob S, Picault N, *et al*. Large tandem duplications affect gene expression, 3D organization, and plant-pathogen response. Genome Res. 2020;30(11):1583–1592. 33033057 10.1101/gr.261586.120 PMC7605254

[92] Mohannath G, Pontvianne F, Pikaard CS. Selective nucleolus organizer inactivation in *Arabidopsis* is a chromosome position-effect phenomenon. Proc. Natl. Acad. Sci. U. S. A. 2016;113(47):13426–13431. 27821753 10.1073/pnas.1608140113 PMC5127337

[93] Chandrasekhara C, Mohannath G, Blevins T, et al. Chromosome-specific NOR inactivation explains selective rRNA gene silencing and dosage control in *Arabidopsis*. Genes Dev. 2016;30(2):177–190. 26744421 10.1101/gad.273755.115 PMC4719308

[94] Picart-Picolo A, Picart C, Picault N, et al. Nucleolus-associated chromatin domains are maintained under heat stress, despite nucleolar reorganization in *Arabidopsis thaliana*. J. Plant Res. 2020;133(4):463–470. 32372397 10.1007/s10265-020-01201-3

[95] Grob S, Schmid MW, Luedtke NW, et al. Characterization of chromosomal architecture in *Arabidopsis* by chromosome conformation capture. Genome Biol. 2013;14(R129):R129. 24267747 10.1186/gb-2013-14-11-r129 PMC4053840

[96] Soppe WJJ, Jasencakova Z, Houben A, *et al*. DNA methylation controls histone H3 lysine 9 methylation and heterochromatin assembly in *Arabidopsis*. EMBO J. 2002;21(23):6549–6559. 12456661 10.1093/emboj/cdf657 PMC136960

[97] Ghaffari R, Cannon EKS, Kanizay LB, et al. Maize chromosomal knobs are located in gene-dense areas and suppress local recombination. Chromosoma. 2013;122(1–2):67–75. 23223973 10.1007/s00412-012-0391-8 PMC3608884

[98] Ananiev EV, Phillips RL, Rines HW. A knob-associated tandem repeat in maize capable of forming fold-back DNA segments: are chromosome knobs megatransposons? Proc Natl Acad Sci U S A. 1998;95(18):10785–10790. 9724782 10.1073/pnas.95.18.10785 PMC27973

[99] Fransz PF, Armstrong S, De Jong JH, *et al*. Integrated cytogenetic map of chromosome arm 4S of *A.* *thaliana*: structural organization of heterochromatic knob and centromere region. Cell. 2000;100(3):367–376. 10676818 10.1016/s0092-8674(00)80672-8

[100] McClintock B. CHROMOSOME MORPHOLOGY IN *ZEA MAYS*. Science. 1929;69(629):629. 17760028 10.1126/science.69.1798.629

[101] Rutowicz K, Lirski M, Mermaz B, *et al*. Linker histones are fine-scale chromatin architects modulating developmental decisions in *Arabidopsis*. Genome Biol. 2019;20(157). DOI:10.1186/s13059-019-1767-3. PMC6685187 31391082

[102] Teano G, Concia L, Carron L, *et al*. Histone H1 protects telomeric repeats from H3K27me3 invasion in *Arabidopsis*. DOI:10.1101/2020.11.28.402172

[103] Moissiard G, Cokus SJ, Cary J, *et al*. MORC family ATPases required for heterochromatin condensation and gene silencing. Science. 2012;336(6087):1448–1451. 22555433 10.1126/science.1221472 PMC3376212

[104] Shaw PJ, Brown JWS. Plant nuclear bodies. Curr. Opin. Plant Biol. 2004;7(6):614–620. 15491908 10.1016/j.pbi.2004.09.011

[105] Ogg SC, Lamond AI. Cajal bodies and coilin–moving towards function. J. Cell Biol. 2002;159(1):17–21. 12379800 10.1083/jcb.200206111 PMC2173504

[106] Love AJ, Yu C, Petukhova NV, *et al*. Cajal bodies and their role in plant stress and disease responses. RNA Biol. 2017;14(6):779–790. 27726481 10.1080/15476286.2016.1243650 PMC5519230

[107] Ding Y, Lozano-Durán R. The Cajal body in plant-virus interactions. Viruses. 2020;12(2):250. 10.3390/v12020250 PMC7077289 32102236

[108] Wang Q, Sawyer I, Sung MH, *et al*. Cajal bodies are linked to genome conformation. Nat Commun. 2016;7(10966):1–17. 10.1038/ncomms10966 PMC4802181 26997247

[109] Molitor AM, Latrasse D, Zytnicki M, *et al*. The *Arabidopsis* hnRNP-Q protein LIF2 and the PRC1 Subunit LHP1 function in concert to regulate the transcription of stress-responsive genes. Plant Cell. 2016;28(9):2197–2211. 27495811 10.1105/tpc.16.00244 PMC5059796

[110] Turck F, Roudier F, Farrona S, *et al*. *Arabidopsis* TFL2/LHP1 specifically associates with genes marked by trimethylation of histone H3 lysine 27. PLoS Genet. 2007;3(e86):e86. 17542647 10.1371/journal.pgen.0030086 PMC1885283

[111] Schoenfelder S, Sugar R, Dimond A, *et al*. Polycomb repressive complex PRC1 spatially constrains the mouse embryonic stem cell genome. Nat. Genet. 2015;47(10):1179–1186. 26323060 10.1038/ng.3393 PMC4847639

[112] Vieux-Rochas M, Fabre PJ, Leleu M, et al. Clustering of mammalian *Hox* genes with other H3K27me3 targets within an active nuclear domain. Proc Natl Acad Sci U S A. 2015;112(15):4672–4677. 25825760 10.1073/pnas.1504783112 PMC4403207

[113] Rosa S, De Lucia F, Mylne JS, *et al*. Physical clustering of *FLC* alleles during Polycomb-mediated epigenetic silencing in vernalization. Genes Dev. 2013;27(17):1845–1850. . 24013499 10.1101/gad.221713.113 PMC3778238

[114] Veluchamy A, Jégu T, Ariel F, *et al*. LHP1 regulates H3K27me3 spreading and shapes the three-dimensional conformation of the *Arabidopsis* genome. PLoS One. 2016;11(7):e0158936. 27410265 10.1371/journal.pone.0158936 PMC4943711

[115] Edelman LB, Fraser P. Transcription factories: genetic programming in three dimensions. Curr. Opin. Genet. Dev. 2012;22(2):110–114. 22365496 10.1016/j.gde.2012.01.010

[116] Di Stefano M, Nützmann H-W, Marti-Renom MA, et al. Polymer modelling unveils the roles of heterochromatin and nucleolar organizing regions in shaping 3D genome organization in *Arabidopsis thaliana*. Nucleic Acids Res. 2021;49(4):1840–1858. 33444439 10.1093/nar/gkaa1275 PMC7913674

[117] Dekker J, Rippe K, Dekker M, et al. Capturing chromosome conformation. Science. 2002;295(5558):1306–1311. 11847345 10.1126/science.1067799

[118] Nagano T, Lubling Y, Stevens TJ, *et al*. Single-cell Hi-C reveals cell-to-cell variability in chromosome structure. Nature. 2013;502(7469):59–64. 24067610 10.1038/nature12593 PMC3869051

[119] Nir G, Farabella I, Pérez Estrada C, *et al*. Walking along chromosomes with super-resolution imaging, contact maps, and integrative modeling. PLoS Genet. 2018;14(12):e1007872. 30586358 10.1371/journal.pgen.1007872 PMC6324821

[120] Serra F, Di Stefano M, Spill YG, *et al*. Restraint-based three-dimensional modeling of genomes and genomic domains. FEBS Lett. 2015;589(20PartA):2987–2995. 25980604 10.1016/j.febslet.2015.05.012

[121] Barrett T, Wilhite SE, Ledoux P, *et al*. NCBI GEO: archive for functional genomics data sets–update. Nucleic Acids Res. 2013;41(D1):D991–5. 23193258 10.1093/nar/gks1193 PMC3531084

[122] Serra F, Baù D, Goodstadt M, *et al*. Automatic analysis and 3D-modelling of Hi-C data using TADbit reveals structural features of the fly chromatin colors. PLoS Comput. Biol. 2017;13(e1005665):e1005665. 28723903 10.1371/journal.pcbi.1005665 PMC5540598

[123] Di Stefano M, Stadhouders R, Farabella I, *et al*. Transcriptional activation during cell reprogramming correlates with the formation of 3D open chromatin hubs. Nat. Commun. 2020;11(2564). DOI:10.1038/s41467-020-16396-1. PMC7244774 32444798

[124] Duan Z, Andronescu M, Schutz K, *et al*. A three-dimensional model of the yeast genome. Nature. 2010;465(7296):363–367. 20436457 10.1038/nature08973 PMC2874121

[125] Trussart M, Yus E, Martinez S, *et al*. Defined chromosome structure in the genome-reduced bacterium *Mycoplasma pneumoniae*. Nat Commun. 2017;8(1). DOI:10.1038/ncomms14665. PMC5344976 28272414

[126] Jhunjhunwala S, Van Zelm MC, Peak MM, *et al*. The 3D structure of the immunoglobulin heavy-chain locus: implications for long-range genomic interactions. Cell. 2008;133(2):265–279. 18423198 10.1016/j.cell.2008.03.024 PMC2771211

[127] Baù D, Marti-Renom MA. Genome structure determination via 3C-based data integration by the Integrative Modeling Platform. Methods. 2012;58(3):300–306. 22522224 10.1016/j.ymeth.2012.04.004

[128] Hua N, Tjong H, Shin H, *et al*. Producing genome structure populations with the dynamic and automated PGS software. Nat. Protoc. 2018;13(5):915–926. 29622804 10.1038/nprot.2018.008 PMC6043163

[129] Li Q, Tjong H, Li X, *et al*. The three-dimensional genome organization of *Drosophila melanogaster* through data integration. Genome Biol. 2017;18(145). DOI:10.1186/s13059-017-1264-5. PMC5576134 28760140

[130] Paulsen J, Liyakat Ali TM, Nekrasov M, *et al*. Long-range interactions between topologically associating domains shape the four-dimensional genome during differentiation. Nat. Genet. 2019;51(5):835–843. 31011212 10.1038/s41588-019-0392-0

[131] Nguyen HQ, Chattoraj S, Castillo D, *et al*. 3D mapping and accelerated super-resolution imaging of the human genome using in situ sequencing. Nat Methods. 2020;17(8):822–832. 32719531 10.1038/s41592-020-0890-0 PMC7537785

[132] Meluzzi D, Arya G. Computational approaches for inferring 3D conformations of chromatin from chromosome conformation capture data. Methods. 2020;181-182:24–34. 31470090 10.1016/j.ymeth.2019.08.008 PMC7044057

[133] MacKay K, Kusalik A. Computational methods for predicting 3D genomic organization from high-resolution chromosome conformation capture data. Brief Funct Genomics. 2020;19(4):292–308. 32353112 10.1093/bfgp/elaa004 PMC7388788

[134] Rosa A, Everaers R, Henikoff S. Structure and dynamics of interphase chromosomes. PLoS Comput. Biol. 2008;4(e1000153):e1000153. 18725929 10.1371/journal.pcbi.1000153 PMC2515109

[135] Jost D, Carrivain P, Cavalli G, et al. Modeling epigenome folding: formation and dynamics of topologically associated chromatin domains. Nucleic Acids Res. 2014;42(15):9553–9561. 25092923 10.1093/nar/gku698 PMC4150797

[136] Fudenberg G, Imakaev M, Lu C, *et al*. Formation of chromosomal domains by loop extrusion. Cell Rep. 2016;15(9):2038–2049. . 27210764 10.1016/j.celrep.2016.04.085 PMC4889513

[137] Grosberg A, Rabin Y, Havlin S, et al. Crumpled globule model of the three-dimensional structure of DNA. EPL. 1993;23(373):373–378.

[138] Rosa A, Becker NB, Everaers R. Looping probabilities in model interphase chromosomes. Biophys J. 2010;98(11):2410–2419. 20513384 10.1016/j.bpj.2010.01.054 PMC2877331

[139] Bystricky K, Heun P, Gehlen L, et al. Long-range compaction and flexibility of interphase chromatin in budding yeast analyzed by high-resolution imaging techniques. Proc Natl Acad Sci U S A. 2004;101(47):16495–16500. 15545610 10.1073/pnas.0402766101 PMC534505

[140] Vettorel T, Grosberg AY, Kremer K. Statistics of polymer rings in the melt: a numerical simulation study. Phys. Biol. 2009;6:025013. 19571364 10.1088/1478-3975/6/2/025013

[141] Halverson JD, Smrek J, Kremer K, et al. From a melt of rings to chromosome territories: the role of topological constraints in genome folding. Rep. Prog. Phys. 2014;77:022601. 24472896 10.1088/0034-4885/77/2/022601

[142] Di Pierro M, Zhang B, Aiden EL, et al. Transferable model for chromosome architecture. Proc. Natl. Acad. Sci. U. S. A. 2016;113(43):12168–12173. 27688758 10.1073/pnas.1613607113 PMC5087044

[143] Jost D, Vaillant C, Meister P. Coupling 1D modifications and 3D nuclear organization: data, models and function. Curr Opin Cell Biol. 2017;44:20–27. 28040646 10.1016/j.ceb.2016.12.001

[144] Di Pierro M, Cheng RR, Lieberman Aiden E, et al. De novo prediction of human chromosome structures: epigenetic marking patterns encode genome architecture. Proc. Natl. Acad. Sci. U. S. A. 2017;114(46):12126–12131. 29087948 10.1073/pnas.1714980114 PMC5699090

[145] Larson AG, Elnatan D, Keenen MM, *et al*. Liquid droplet formation by HP1α suggests a role for phase separation in heterochromatin. Nature. 2017;547(7662):236–240. 28636604 10.1038/nature22822 PMC5606208

[146] Strom AR, Emelyanov AV, Mir M, *et al*. Phase separation drives heterochromatin domain formation. Nature. 2017;547(7662):241–245. . 28636597 10.1038/nature22989 PMC6022742

[147] Nora EP, Lajoie BR, Schulz EG, *et al*. Spatial partitioning of the regulatory landscape of the X-inactivation centre. Nature. 2012;485(7398):381–385. 22495304 10.1038/nature11049 PMC3555144

[148] Benedetti F, Dorier J, Burnier Y, et al. Models that include supercoiling of topological domains reproduce several known features of interphase chromosomes. Nucleic Acids Res. 2014;42(5):2848–2855. 24366878 10.1093/nar/gkt1353 PMC3950722

[149] Sanborn AL, Rao SSP, Huang S-C, *et al*. Chromatin extrusion explains key features of loop and domain formation in wild-type and engineered genomes. Proc Natl Acad Sci U S A. 2015;112(47):E6456–65. 26499245 10.1073/pnas.1518552112 PMC4664323

[150] Brackley CA, Johnson J, Michieletto D, *et al*. Nonequilibrium chromosome looping via molecular slip links. Phys. Rev. Lett. 2017;119(138101). DOI:10.1103/PhysRevLett.119.138101. 29341686

[151] Brackley CA, Johnson J, Kelly S, et al. Simulated binding of transcription factors to active and inactive regions folds human chromosomes into loops, rosettes and topological domains. Nucleic Acids Res. 2016;44(8):3503–3512. 27060145 10.1093/nar/gkw135 PMC4856988

[152] Barbieri M, Chotalia M, Fraser J, *et al*. Complexity of chromatin folding is captured by the strings and binders switch model. Proc Nat Acad Sci USA. 2012;109(40):16173–16178. 22988072 10.1073/pnas.1204799109 PMC3479593

[153] Scialdone A, Cataudella I, Barbieri M, et al. Conformation regulation of the X chromosome inactivation center: a model. PLoS Comput. Biol. 2011;7(e1002229):e1002229. 22046112 10.1371/journal.pcbi.1002229 PMC3203058

[154] Barbieri M, Xie S, Torlai Triglia E, *et al.* Active and poised promoter states drive folding of the extended *HoxB* locus in mouse embryonic stem cells. Nat Struct Mol Biol. 2017;24:515–524. 10.1038/nsmb.3402 28436944

[155] Heger P, Marin B, Bartkuhn M, et al. The chromatin insulator CTCF and the emergence of metazoan diversity. Proc Natl Acad Sci U S A. 2012;109(43):17507–17512. 23045651 10.1073/pnas.1111941109 PMC3491479

[156] Bolaños-Villegas P, De K, Pradillo M, et al. In favor of establishment: regulation of chromatid cohesion in plants. Front Plant Sci. 2017;8(846). DOI:10.3389/fpls.2017.00846 PMC5440745 28588601

[157] Zhang J, Zhang B, Su H, et al. Molecular mechanisms of homologous chromosome pairing and segregation in plants. J Genet Genomics. 2014;41(3):117–123. 24656232 10.1016/j.jgg.2013.12.003

[158] De Nooijer S, Wellink J, Mulder B, et al. Non-specific interactions are sufficient to explain the position of heterochromatic chromocenters and nucleoli in interphase nuclei. Nucleic Acids Res. 2009;37(11):3558–3568. 19359359 10.1093/nar/gkp219 PMC2699506

[159] Cuacos M, Franklin FCH, Heckmann S. Atypical centromeres in plants—what they can tell us. Front Plant Sci. 2015;6. DOI:10.3389/fpls.2015.00913 PMC4620154 26579160

